# Effect of nitric oxide on postoperative acute kidney injury in patients who underwent cardiopulmonary bypass: a systematic review and meta-analysis with trial sequential analysis

**DOI:** 10.1186/s13613-019-0605-9

**Published:** 2019-11-21

**Authors:** Jie Hu, Stefano Spina, Francesco Zadek, Nikolay O. Kamenshchikov, Edward A. Bittner, Juan Pedemonte, Lorenzo Berra

**Affiliations:** 1 Department of Anesthesia, Critical Care and Pain Medicine, Massachusetts General Hospital, 55 Fruit Street, Boston, MA USA; 2 Department of Critical Care Medicine, Chinese PLA General Hospital, 28 Fuxing Road, Beijing, China; 3 Department of Anesthesia and Critical Care Medicine, Cardiology Research Institute, Tomsk National Research Medical Center, Russian Academy of Sciences, 111a Kievskaya St., Tomsk, 634012 Russia; 4 División de Anestesiología, Escuela de Medicina, Pontificia Universidad Católica de Chile, Santiago, Chile

**Keywords:** Nitric oxide, Cardiopulmonary bypass, Acute kidney injury, Meta-analysis, Trial sequential analysis

## Abstract

**Background:**

The effect of nitric oxide (NO) on renal function is controversial in critical illness. We performed a systematic meta-analysis and trial sequential analysis to determine the effect of NO gas on renal function and other clinical outcomes in patients requiring cardiopulmonary bypass (CPB). The primary outcome was the relative risk (RR) of acute kidney injury (AKI), irrespective of the AKI stage. The secondary outcome was the mean difference (MD) in the length of ICU and hospital stay, the RR of postoperative hemorrhage, and the MD in levels of methemoglobin. Trial sequential analysis (TSA) was performed for the primary outcome.

**Results:**

54 trials were assessed for eligibility and 5 studies (579 patients) were eligible for meta-analysis. NO was associated with reduced risk of AKI (RR 0.76, 95% confidential interval [CI], 0.62 to 0.93, *I*^2^ = 0%). In the subgroup analysis by NO initiation timing, NO did not decrease the risk of AKI when started at the end of CPB (RR 1.20, 95% CI 0.52–2.78, *I*^2^ = 0%). However, NO did significantly reduce the risk of AKI when started from the beginning of CPB (RR 0.71, 95% CI 0.54–0.94, *I*^2^ = 10%). We conducted TSA based on three trials (400 patients) using KDIGO criteria and with low risk of bias. TSA indicated a CI of 0.50–1.02 and an optimal information size of 589 patients, suggesting a lack of definitive conclusion. Furthermore, NO does not affect the length of ICU and hospital stay or the risk of postoperative hemorrhage. NO slightly increased the level of methemoglobin at the end of CPB (MD 0.52%, 95% CI 0.27–0.78%, *I*^2^ = 90%), but it was clinically negligible.

**Conclusions:**

NO appeared to reduce the risk of postoperative AKI in patients undergoing CPB. Additional studies are required to ascertain the finding and further determine the dosage, timing and duration of NO administration.

## Background

Acute kidney injury (AKI) is a multifactorial and common complication in patients undergoing cardiopulmonary bypass (CPB), as it occurs in 30–70% of patients [1–4]. CPB leads to a decrease in the bioavailability of vascular nitric oxide (NO), both because of NO scavenging (via deoxygenation reaction in the presence of intravascular hemolysis [5, 6]) and through a reduction in NO synthesis (in the presence of ischemia/reperfusion injury, acute inflammatory reaction and endothelial dysfunction [7]). In particular, hemolysis generated during the CPB has been demonstrated to be a pivotal contributor to the increased risk of perioperative AKI in patients undergoing CPB [6].

NO regulates vascular tone and distal blood perfusion while also acting as an anti-inflammatory and anti-thrombotic mediator [8]. NO gas is traditionally used for the treatment of acute exacerbation of pulmonary hypertension [9] and pediatric hypoxemic respiratory failure [10]. At present, NO has been tested in several randomized trials for its protective role in pulmonary hypertension and myocardial injury in patients undergoing CPB [11–13]. More recently, NO has been shown to protect against AKI and chronic renal disease, either by reprogramming metabolism [14] or via homeostatic regulation of renal hemodynamics and α1-adrenoreceptor sensitization [15]. Administration of NO gas was associated with benefit of lowering plasma NO consumption in the presence of hemolysis [16, 17]. Lei et al. conducted a single-center randomized controlled trial (RCT) and found that NO delivered from the beginning of CPB could reduce the risk of AKI and lower NO consumption in plasma [18], a finding which was confirmed by a recently completed randomized trial [19]. On the contrary, in a meta-analysis, Ruan et al. showed that NO therapy was associated with renal dysfunction, especially in critically ill patients with acute respiratory distress syndrome (ARDS) [20].

We hypothesized that the effect of NO on kidney function might be disease specific, as two of the RCTs in the meta-analysis of Ruan et al. [20] showed that NO administration started from the end of CPB in cardiac surgery patients [21, 22] had no adverse effects on renal function. Thus, a careful analysis of the effect of NO gas on renal function in cardiac surgery is warranted.

We performed a meta-analysis aiming to ascertain the effect of NO therapy on renal function in patients undergoing CPB and to further investigate whether the effect of NO varies between different initiation time points of such therapy.

## Methods

This systematic review and meta-analysis was registered on PROSPERO (NO. CRD42019125948) based on the Preferred Reporting Items for Systematic Reviews and Meta-Analyses (PRISMA) guidelines [23].

### Search strategy

Two trained investigators (J.H. and J.P.) independently searched pertinent studies in PubMed, Cochrane Central Register of Controlled Trials (CENTRAL), Embase, Web of Science, and Clinicaltrial.gov from inception through December 2018, with the help of a librarian from the Countway Library at Harvard Medical School. Details of our search strategy are described in Additional file 1 (data not shown).

### Study selection

Two investigators (J.H., J.P.) independently searched if the eligible studies met the following PICOS criteria: (1) population: patients undergoing CPB; (2) intervention: NO delivery either from the beginning of CPB or at the end of CPB; (3) comparison intervention: placebo or no therapy; (4) outcome: the relative risk of postoperative AKI; and (5) study design: randomized controlled trials (RCTs).

There were no restrictions on the dose or time of NO administration. We included unpublished trials only if trial data and methodological descriptions were provided either in written form or could be retrieved from the trial authors. No language restriction was enforced. The exclusion criteria included crossover trials and studies not reporting the renal outcome.

### Data extraction and study characteristics

Two authors (J.H. and J.P.) independently assessed the selected studies for the final analysis, with disagreements resolved by discussion and, if needed, via a third author (LB), who acted as an adjudicator. A standardized recording form was used for data extraction.

The primary outcome was the risk of AKI, irrespective of the AKI stage. The secondary outcome was as follows: the length of ICU and hospital stay, the risk of postoperative hemorrhage (i.e., requiring blood transfusion after the operation or reoperation), and the levels of methemoglobin (MetHb) at the end of CPB.

### Assessment of risk of bias

Quality assessment of the screened studies was performed using the Cochrane Collaboration tool [24] by two independent authors (J.H. and J.P.), and discrepancies were resolved by consensus. The following domains were evaluated individually and graded as “low risk”, “high risk”, or “unclear risk”: random sequence generation, allocation concealment, blinding of participants and personnel, blinding of outcome assessment, incomplete outcome data, selective outcome reporting, and other bias.

### Data analysis and synthesis

We calculated the pooled risk ratios (RR) and 95% confidence intervals (CI) for the binary outcome of AKI, and postoperative hemorrhage using the Mantel–Haenszel method with the random-effects model. We estimated the mean difference (MD) and 95% CI for continuous outcomes of the length of ICU and hospital stay and the levels of MetHb using the inverse variance method with the random-effects model. If continuous variables were expressed as a median and interquartile range, the mean and standard deviation were computed based on the median, interquartile range, and sample size as described elsewhere [25].

Considering that the inclusion of fewer than ten studies in meta-analysis causes low statistical power for detecting funnel plot asymmetry [26], we assessed the risk of publication bias by visual examination of the funnel plot. To assess and adjust for potential publication bias in the meta-analysis, we performed the trim-and-fill test [27]. The amount of heterogeneity was assessed by both Cochran’s Q test and the *I*^2^ statistic [28].

We performed a subgroup analysis to investigate whether the effect of NO on AKI varied based on the timing of NO therapy initiation, i.e., starting from the beginning of CPB or at the end of CPB (immediately before the weaning of CPB).

We performed sensitivity analyses to determine the robustness of the effect size using different data analysis methods. First, we used the Peto method, since it may be the least biased in the presence of sparse data and imbalance of sample size within trials [29]. Second, to reduce the intrinsic heterogeneity among studies based on the outcome measure, we omitted the two studies that did not use the KDIGO criteria in AKI and repeated the evaluation.

We conducted trial sequential analysis (TSA) to quantify the statistical reliability of data in the cumulative meta-analysis and adjusted significance levels for the risk of random errors due to repetitive testing on accumulating data [30]. For this analysis, we employed a random-effects model using the DerSimonian–Laird Method and included trials with low risk of bias and that used the KDIGO criteria for AKI diagnosis. We intended to maintain an overall 5% risk of a type I error and a power of 80%. For the calculation of the required IS, we anticipated an intervention effect of a 30% relative risk reduction (RRR) as suggested by Lei et al. [18]. We used a control event proportion calculated from the actual meta-analysis. We calculated TSA-adjusted CI (alpha spending) for effect size.

Statistical analyses were conducted using Review Manager v.5.3 (The Nordic Cochrane Centre, The Cochrane Collaboration, Copenhagen, Denmark), Trial Sequential Analysis v.0.9.5.10 beta (Copenhagen Trial Unit, Centre for Clinical Intervention Research, Rigshospitalet, Copenhagen, Denmark, available from http://www.ctu.dk/tsa) and R v.1.1.456 (R Core Team, R Foundation for Statistical Computing, Vienna, Austria) with the “metafor” and “meta” packages.

## Results

### Literature search and study characteristics

**Fig. 1:**
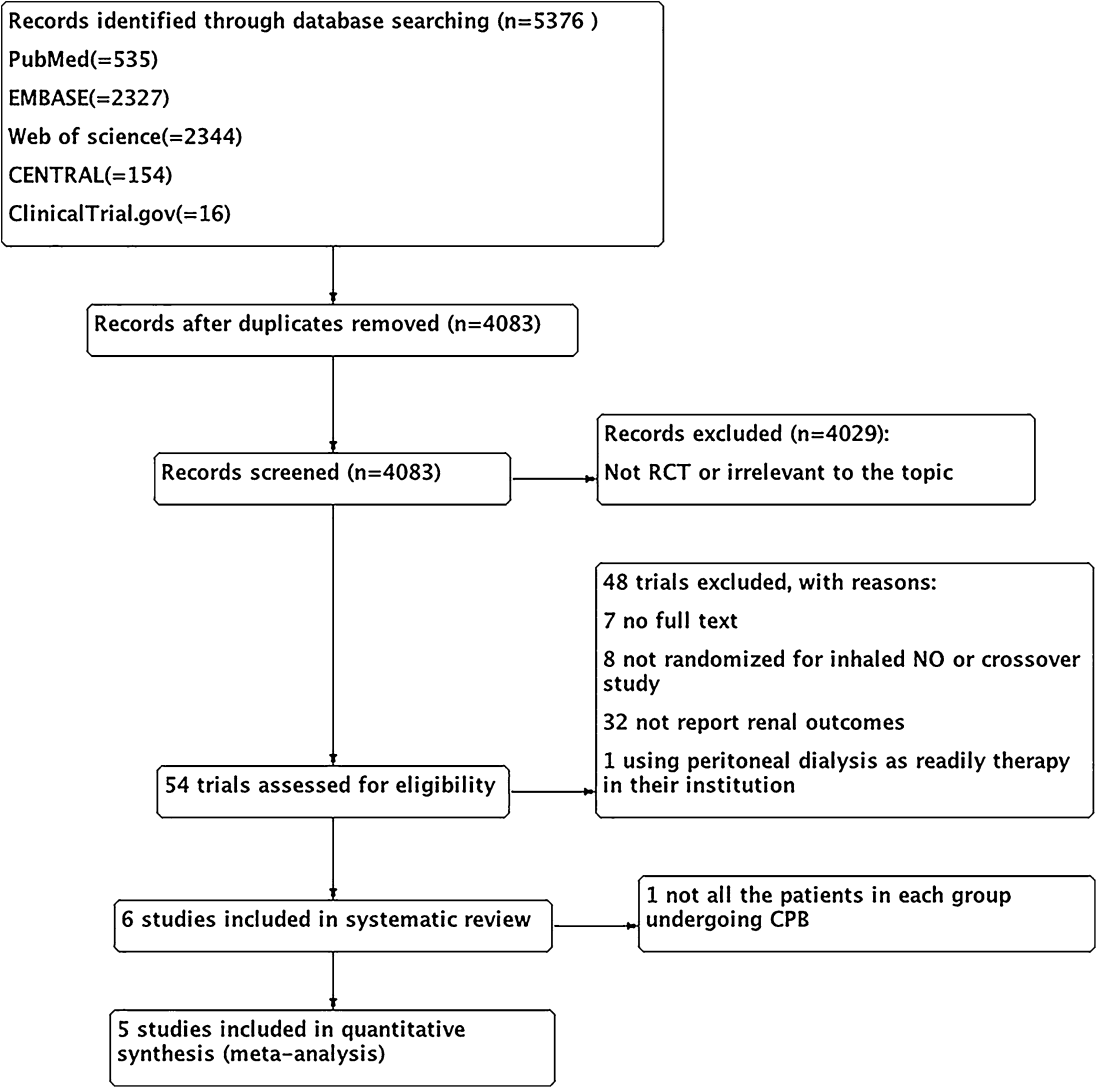
Flow chart of the systematic review and meta-analysis in the present study

Through the electronic search, 5376 citations were identified. After excluding 1293 duplicates, 4083 studies were chosen for further evaluation. Through reading the titles and abstracts, 4029 ineligible studies were excluded, and 54 studies were identified as potentially eligible for inclusion and were evaluated by reading the full text. Forty-eight studies were excluded due to lack of full text, not randomized for NO therapy or crossover studies, not reporting renal outcomes, and using peritoneal dialysis as readily therapy. Finally, six studies were eligible for systematic review [18, 19, 21, 22, 31, 32]. Among the six included studies, one study was ultimately excluded, because not all the patients in NO and control groups underwent CPB [32]. One study was completed recently and relevant data were obtained from the primary investigator (N. Kamenshchikov) [19]. Figure 1 shows the process of literature selection and reasons for study exclusion.

**Fig. 2:**
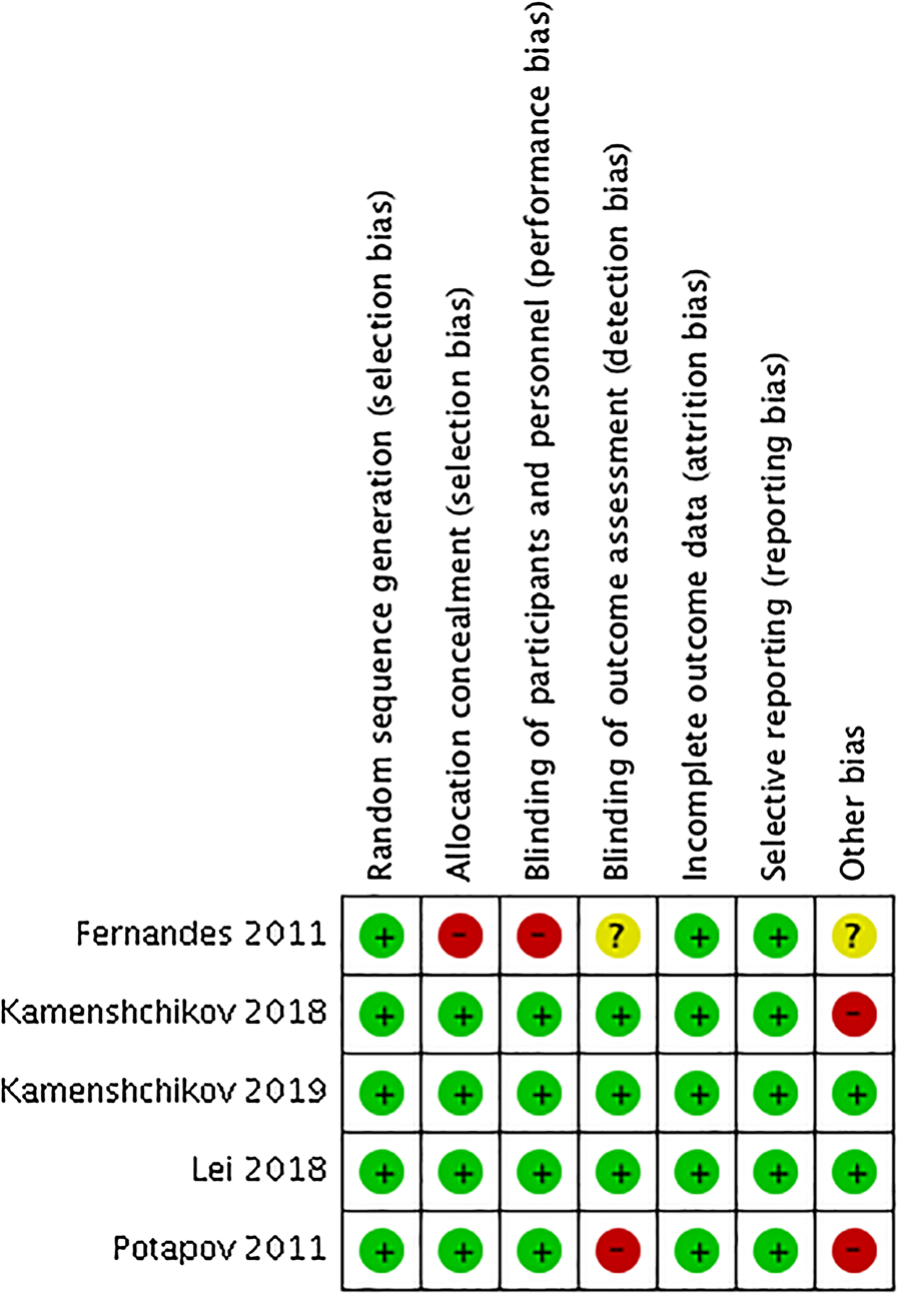
Risk of bias summary for each included trial. Red circles indicate high risk. Green circles indicate low risk. Yellow circles indicate unclear risk

The characteristics of the included studies are summarized in Table 1. These studies vary in AKI definition and in the time of initiation of NO therapy. Two studies started NO therapy immediately before CPB weaning [21, 22] and defined renal dysfunction as urine output less than 0.3 ml/h [21] or need for renal replacement treatment (RRT) [22], respectively. Three studies delivered NO from the beginning of CPB and defined AKI by the KDIGO criteria [18, 19, 31]. Detailed information of all included studies is shown in Table 1, and quality evaluation is shown in Fig. 2 and Additional file 2 (data not shown).

**Table 1:** Details of the included randomized controlled trials

Study (year)	Population	The protocol of NO therapy	Comparison	Definition of AKI	Duration of CPB (min)		No. of AKI/no. of cases	
					NO	Control	NO	Control
Potapov (2011)	Adults, LVAD placement	40 ppm within 48 h, through inhalation^#^	Placebo	Need for RRT	NA	NA	10/73	8/77
Fernandes (2011)	Adults, mitral stenosis and severe pulmonary hypertension	10 ppm within 48 h, through inhalation^#^	Oxygen	Urine output < 0.3 ml/kg/h	88 ± 31^#^	94 ± 34^#^	0/14	1/15
Lei (2018)	Adults, multiple valve replacement surgery, mostly due to rheumatic fever	80 ppm within 24 h,through CPB and inhalation	Placebo	KDIGO criteria (SCr only)	138 (122;159)^$&^	134 (114;154)^&^	58/117	81/127
Kamenshchikov (2018)	Adults, CABG	40 ppm through CPB	Standard CPB	KDIGO criteria (SCr only)*	110 (85.8;137)^&^	116 (88.8;129.5)^&^	1/30	3/30
Kamenshchikov (2019)	Adults, CABG, valve surgery, surgical reconstruction of the left ventricle	40 ppm through CPB	Standard CPB	KDIGO criteria (SCr and urine output)	118 (95.5;167.5)^&^	119 (91.7;130.4)^&^	10/48	20/48

*LVAD* left ventricular assist device, *AKI* acute kidney injury, *CABG* coronary artery bypass grafting, *CPB* cardiopulmonary bypass; placebo, an equivalent concentration of nitrogen; KDIGO criteria* only monitoring for 2 days after operation, *RRT* renal replacement therapy; through inhalation ^#^ started NO administration immediately at the discontinuation of CPB; ^$^ *p* = 0.048; ^#^ mean ± SD; ^&^ median (interquartile range)

### Quantitative data synthesis

**Fig. 3:**
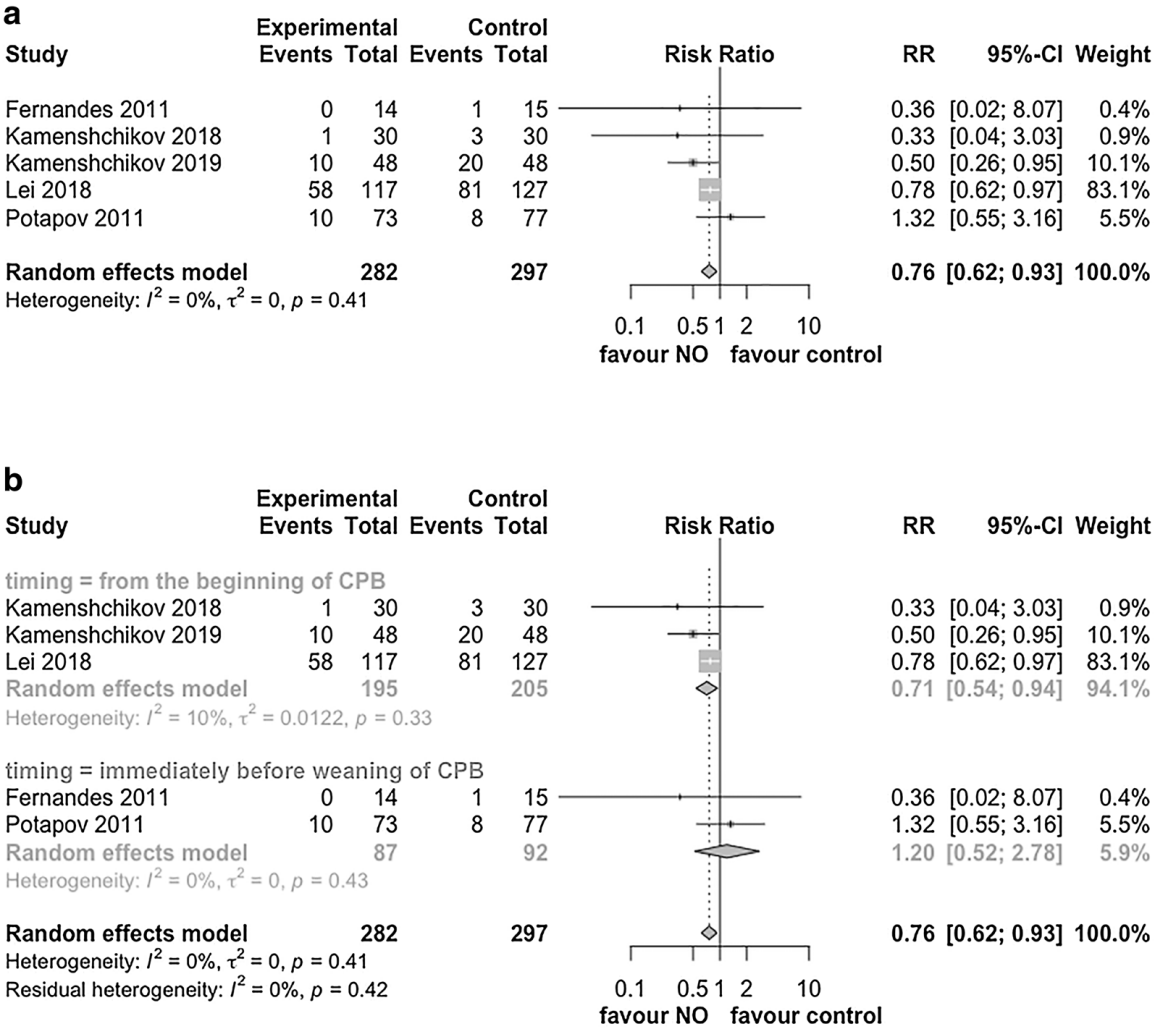
The relative risk of postoperative AKI. **a** Forest plot of the risk of AKI irrespective of the AKI stage in included trials. **b** Forest plot of subgroup analysis by the timing of NO initiation. *RR* risk ratio, *CI* confidential interval, *AKI* acute kidney injury, *NO* nitric oxide

For the primary outcome of AKI irrespective of the stage, the pooled effect showed that NO therapy significantly reduced the risk of AKI with RR of 0.76 (95% CI 0.62–0.93, *I*^2^ = 0%, *p* = 0.008, Fig. 3a).

To evaluate the impact of the timing of NO therapy initiation on the development of AKI, we performed a subgroup analysis. NO therapy did not appear to decrease the risk of AKI when it started immediately before the weaning of CPB (RR, 1.20, 95% CI 0.52–2.78, *p* = 0.67, *I*^2^ = 0%, Fig. 3b). Conversely, NO did appear to significantly reduce the risk of AKI when administered from the beginning of CPB (RR, 0.71, 95% CI 0.54–0.94, *p* = 0.02, *I*^2^ = 10%, Fig. 3b).

**Fig. 4:**
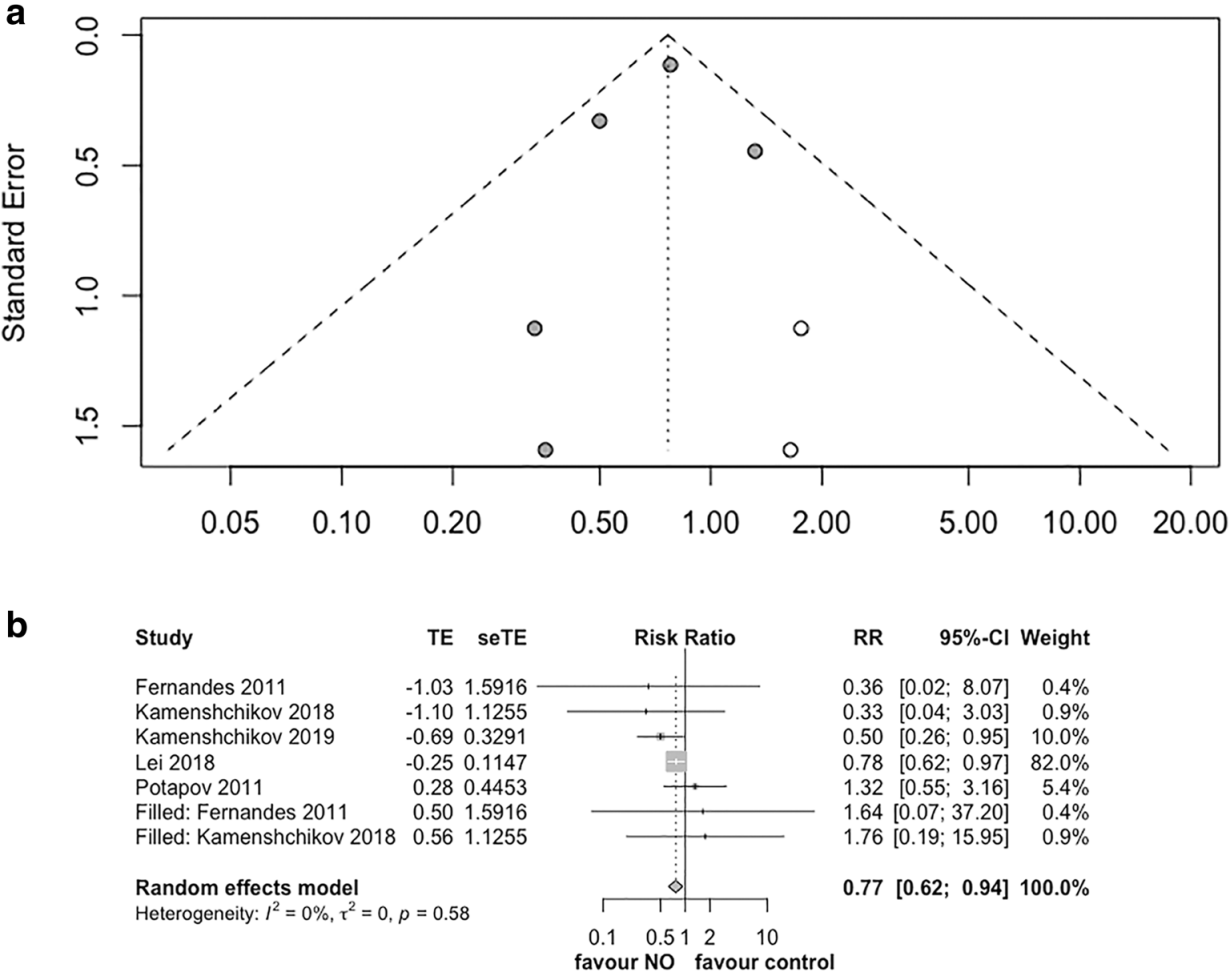
Trim-and-fill test for the primary outcome. **a** Funnel plot of the trim-and-fill test. Solid dots indicate included trials. Blanks dots indicated filled unpublished studies. **b** Forest plot of the trim-and-fill test for the primary outcome. *RR* risk ratio, *CI* confidential interval, *AKI* acute kidney injury, *NO* nitric oxide

The funnel plot based on the primary outcome (Additional file 3, data now shown) showed asymmetry on visual inspection. This suggests that the pooled effect from the current data might overestimate the improvement of NO on renal function. We performed the trim-and-fill test to adjust the publication bias. It confirmed the benefit of NO on renal function with adjusted RR of 0.77 (95% CI 0.62–0.94, *p* = 0.01, *I*^2^ = 0%, Fig. 4a, b).

Sensitivity analyses were performed to evaluate the influence of data synthesis methods on the estimate of the summary effect. First, to evaluate the potential impact of the sparse data and imbalance of sample size, we pooled the effect sizes by the Peto method and found that it was similar to that obtained by the primary analysis (Table 2, Additional file 4, data not shown). Second, the three studies that had low risk of bias and reported AKI using KDIGO criteria [18, 19, 31] were included in a sensitivity analysis. Data also corroborated the beneficial effect of NO on renal function (RR, 0.71, 95% CI 0.54–0.94, *p* = 0.02, *I*^2^ = 10%, Additional file 5, data not shown).

**Table 2:** Sensitivity analyses

Outcome measures	Number of studies (number of patients)	Statistical model	Effect size (95% CI)	*p* value (test for effect)	Heterogeneity (*I*^2^), %
AKI	5 (597)	RR random effects	0.76 (0.62–0.93)	0.008	0
		OR random effects	0.58 (0.38–0.90)	0.015	6
		OR Peto	0.58 (0.37–0.92)	0.019	12
AKI	3 (400)	RR random effects	0.71 (0.54–0.94)	0.018	10

*AKI* acute kidney injury, *RR* risk ratio, *OR* odds ratio

**Fig. 5:**
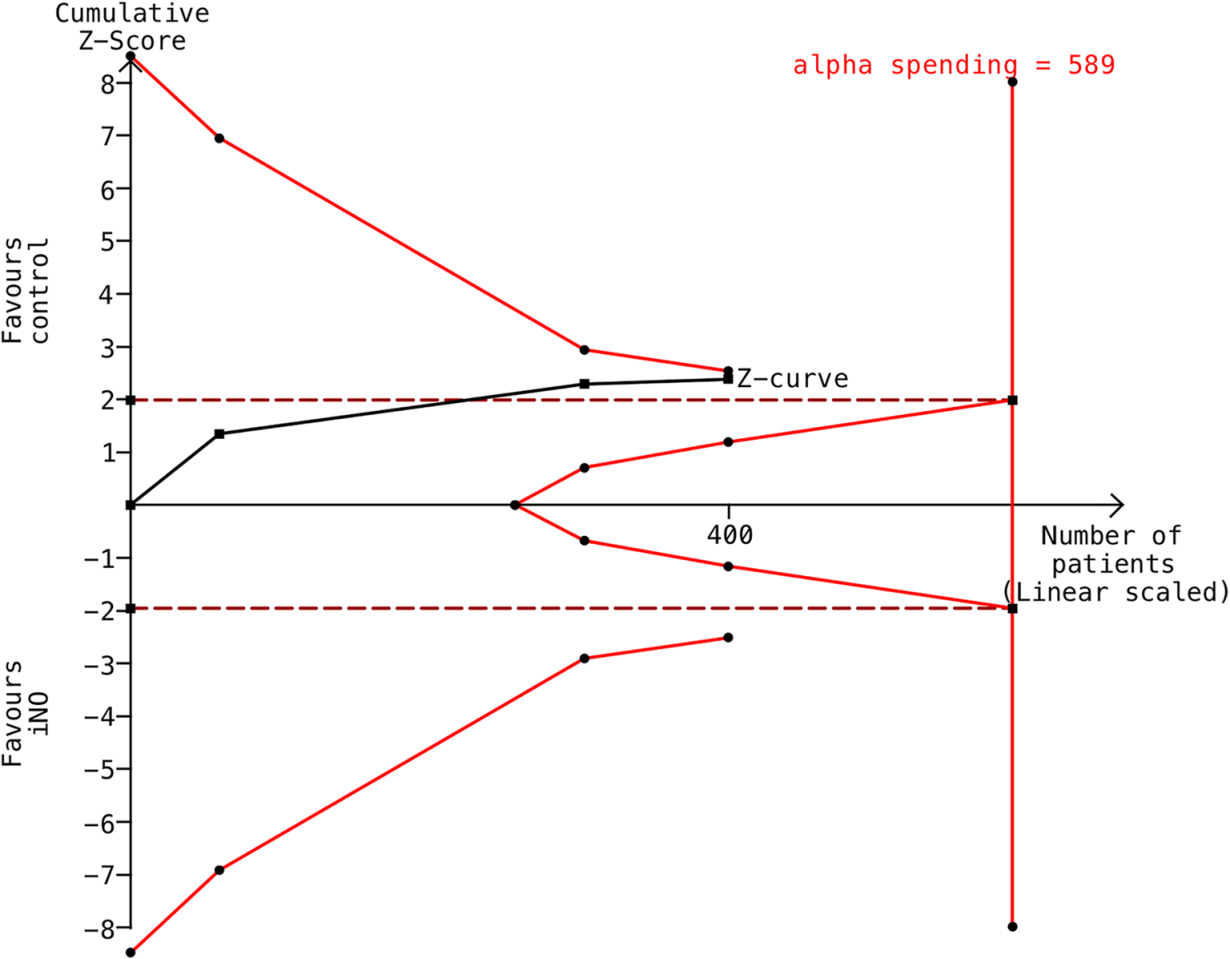
Trial sequential analysis (TSA) for the primary outcome. The TSA of the included trials (black square fill icons) shows that the cumulative *Z* curve did cross the traditional boundary (wine red dotted line) but did not cross the trial sequential monitoring boundary (red full line) for futility or reach the required information size (*n* = 589). *X*-axis: the number of patients randomized; *y*-axis: the cumulative *z* score; horizontal wine red dotted lines: conventional boundaries (upper for benefit, *z* score = 1.96, lower for harm, *z* score = − 1.96, two-sided, *p* = 0.05); oblique red lines with black full circle icons: trial sequential monitoring boundaries; oblique black line with black full square icons: *Z* curve; and vertical red straight line with circles: required information size. The diversity-adjusted required information size (589 participants) was based on a relative risk reduction of 30%, an alpha of 5%, a beta of 20%, and an event proportion of 50.7% in the control arm. The red cumulative *Z* curve was constructed using a random-effects model with the DerSimonian–Laird Method

To decrease the risk of random errors due to sparse data or repetitive testing and calculate the optimal information size for this meta-analysis, we performed TSA based on three trials that used KDIGO criteria and were considered to have a low risk of bias (400 patients). Although the cumulative *Z* curve crossed the traditional boundary for statistical significance, it did not cross the TSA monitoring boundary or reach the information size (Fig. 5). Based on this analysis, the optimal information size was found to be 589 patients for risk of AKI. The alpha spending adjusted CI was 0.50–1.02 (*I*^2^ = 10%, *D*^2^ = 44%) based on 30% RRR (from a baseline event rate of 50.8%).

For the secondary outcomes, there appeared to be no benefit of NO on the length of ICU and hospital stay, with MD of − 0.09 day (95% CI − 0.22–0.03, *p* = 0.15, *I*^2^ = 0%, Additional file 6, data not shown) and MD of − 0.51 day (95% CI − 1.17–0.16, *p* = 0.14, *I*^2^ = 27%, Additional file 7, data not shown), respectively.

The effect of NO administration during CBP on hemorrhage was examined as a potential complication. NO administration did not increase the risk of postoperative hemorrhage in patients undergoing CPB with RR 0.79 (95% CI 0.22–2.83, *p* = 0.71, *I*^2^ = 35%, Additional file 8, data not shown).

As safety outcome, levels of MetHb at the end of CPB were also evaluated. Since Lei et al. measured the levels of MetHb at different time points, which peaked at the end of the CPB [18], the levels of MetHb at the end of CPB were studied and showed NO increased levels of MetHb with MD of 0.52% (95% CI 0.27–0.78%, *p* < 0.001, *I*^2^ = 90%) (Additional file 9, data not shown).

## Discussion

In this study, we investigated the effect of NO therapy on the risk of postoperative AKI in patients undergoing CPB. Overall, NO reduced the incidence of AKI in patients undergoing CPB. Although visual inspection of the funnel plot suggested publication bias, the trim-and-fill test still confirmed a beneficial effect of NO administration on the development of AKI. Sensitivity analyses also revealed consistent effect estimates for the primary outcome. However, TSA analysis suggests that further studies are required to achieve a firm conclusion. NO supplementation neither reduced the length of the hospital or ICU stay nor increased the risk of postoperative hemorrhage. NO therapy slightly increased the level of MetHb, which was clinically negligible and always below safety thresholds.

The nephrotoxicity of NO therapy has emerged since the aforementioned meta-analysis was published [20], which suggested that NO impaired renal function in critical illness settings. To interpret our results in the contest of the present literature, we asked two questions.

Our first question is: why has NO therapy shown differing effects on renal function in patients undergoing CPB as compared to patients with ARDS [20]? Several mechanisms may explain the contradictory scenario. CPB contributes to the development of AKI through multiple mechanisms including [1] hypoperfusion (microcirculatory), [2] ischemia/reperfusion injury, [3] hemodilution, [4] pro-inflammatory response, and most importantly, and [5] intravascular hemolysis [33]. Hemolysis strongly correlates to increased plasma NO depletion leading to a decrease of NO bioavailability [6, 34]. Nitric oxide is a potent endogenous vasodilator released by endothelial cells and its depletion leads to vasoconstriction, and ultimately to reduce organ perfusion [17, 35, 36]. The renal protective effects of NO gas might be twofold. On one hand, the administration of NO may act as the replenishment of NO storage in the presence of NO depletion due to hemolysis. On the other hand, NO gas might generate plasma NO metabolites that are protective against ischemia–reperfusion injury [37]. In this context, administration of therapeutic NO has shown promising properties by lowering vascular NO depletion [16], which could explain why breathing 40 parts per million (ppm) of NO markedly increased renal blood flow, glomerular filtration rate, and urine flow in a swine model of phenylephrine-induced hypertension [38].

In comparison, there is no obvious NO deficiency in ARDS. Instead, intrapulmonary NO generation due to inducible NO synthase was found in an experimental model of endotoxemia-induced ARDS and finally proved to be involved in the development of ARDS [39]. Moreover, Ruan et al. found that the duration of NO administration was longer in ARDS studies (> 7 days) [20]. Thus, prolonged NO therapy could induce plasma NO redundancy and in turn [1] induce tubular apoptosis [40], [2] produce reactive nitrogen species, such as nitrogen dioxide (NO_2_) [41], and create a pro-inflammatory response leading to renal vasoconstriction and injury [20]. Based on present literature, we suggested that the effect of NO on renal function might be disease specific.

Our second question is: does the effect of NO on renal function vary by the timing of initiation? To answer to this question, one should consider the differences in renal dysfunction definition adopted in the five studies included in our meta-analysis. The first two studies in which NO was administrated at the end of CPB, renal dysfunction was defined as severe AKI (i.e., urine output < 0.3 ml/kg/h [21] or need for renal replacement therapy [22]). In the following three studies [18, 19, 31] in which NO gas was delivered at the beginning of CPB, renal dysfunction followed KDIGO criteria. Due to the dissimilar definitions of renal injury, we are unable to make a definitive conclusion on the renal protective properties of NO delivery when started at the end of CPB. Indeed, based on biochemical [6, 33, 42–44] and hemodynamic studies [45–50] discussed below, it is plausible that late delivery of NO might not protect the kidney function.

Red blood cells (RBCs) are damaged when passing through the CPB circuit [33] and thus releasing free hemoglobin (Hb) into the circulation [42]. High levels and prolonged duration of hemolysis can scavenge the NO produced by the endothelial cells, with deleterious effects on vascular endothelium and renal tubular cells [33, 43]. Vermeulen et al. showed that patients with postoperative AKI had higher levels of plasma Hb at the end of CPB, as compared to patients without AKI [6]. Meanwhile, circulating Hb also promotes oxidative stress and degrades to release free heme and heme iron, triggering the activation of the immune response through the innate pathway (e.g., TLR-4 pathway) [43]. Based on current knowledge, drugs that could prevent endothelial dysfunction by scavenging free Hb and reactive oxygen species could be a future therapeutic option. If NO is delivered at the beginning of CPB, it can oxidize heme iron to the ferric state and transform hemoglobin to MetHb [44] before the blood is reinfused in the patient. As a result, plasma availability of NO is preserved; blood flow to the organs is maintained, thereby lowering the risk of perioperative AKI. If NO is delivered at the end of CPB, especially when prolonged, hemolysis-associated consequences have already started and subsequently induce AKI via systemic vascular injury, inflammatory cascade, and oxidative stress.

Moreover, hemolysis-induced NO consumption increased pulmonary vascular resistance [34], thereby leading to right heart dysfunction and subsequent postoperative AKI [51]. However, administration of NO at the end of CPB [21, 22, 45–48] or even later in the intensive care unit [49, 50] showed no effect on pulmonary hypertension. Among the five studies included in our meta-analysis, Fernandes et al. demonstrated that patients receiving inhaled NO had decreased pulmonary vascular resistance but a similar pulmonary artery systolic pressure, compared to the control group [21]. Potapov et al. found that inhaled NO did not affect right heart function in terms of risk of right heart disease, pulmonary vascular resistance index, and incidence of central venous pressure more than 16 cmH_2_O [22]. Furthermore, in other clinical trials assessing effect of NO on pulmonary hypertension or heart function among patients requiring CPB, administration of NO at the end of CPB [45–48] or in the intensive care unit [49, 50] did not affect the mean pulmonary artery pressure, and a subsequent meta-analysis also confirmed the aforementioned phenomenon [52]. Further hemodynamic studies should determine whether NO delivered at the beginning of CPB could improve right heart function and pulmonary hypertension, to better interpret the potential mechanism of NO in renal protection.

Although NO appears to improve renal function in patients requiring CPB, possible adverse effects need to be monitored during gas delivery. Hemorrhage is one of the common concerns of NO treatment in cardiac surgery patients undergoing CPB. NO ha been shown to inhibit platelet activation in in vitro studies [53] and potentially prolong bleeding time. However, no clinical trials showed an increased risk of bleeding when NO was delivered in cardiac surgery. We confirmed those findings with the present meta-analysis.

The monitoring of MetHb levels in the blood is warranted during NO delivery. MetHb (Fe^3+^) has a lower capacity to bind oxygen compared to oxy-hemoglobin (Fe^2+^), which may lead to decreased delivery of oxygen to the peripheral tissues and, consequent, tissue hypoxia. It is generally accepted that blood MetHb levels in healthy individuals is less than 2% of the hemoglobin [54]. Cyanosis is present when the MetHb levels approach 15–20% [54]. Our meta-analysis showed a slight increase in blood MetHb level in the NO group and never exceeded 10% in any patient according to the included RCTs [18, 19]. Meanwhile, Potapov et al. reported that levels of MetHb were not higher in NO group compared with the control group [22] and Kamenshchikov et al. found out that all the patients in their study had levels of MetHb less than 0.5% during CPB [31].

This study has some limitations. First, the small number of studies included in our meta-analysis may have reduced the statistical power of the analysis. To balance our interpretation, we performed trial sequential analysis, which accounts for the type I, and type II errors, using widely accepted, methods for adjusting thresholds for significance in randomized clinical trials when the required sample size has not been reached [30].

Second, although the level of statistical heterogeneity was very low in our analyses, the heterogeneity in other independent variables including AKI diagnosis criteria, duration and dosage of NO therapy, and time of CPB should not be overlooked. Over the past decades, the diagnostic criteria for AKI and AKI stage definitions have changed, i.e., decades ago common medical terminology referred to acute renal failure to describe kidney injury in acute settings, subsequently, the RIFLE criteria introduced the term AKI [55], the AKIN perfected the definition of AKI [56] and more recently those definitions have been updated in KDIGO classification [57]. Thus, it is not surprising that many meta-analyses and epidemiological studies reported in the literature include heterogeneous AKI definitions [20, 58].

Finally, some competing endpoints, such as 28- or 90-day mortality, the impact of different dose and duration of NO on renal function, and the right heart function were not reported in the present study.

## Conclusion

In our meta-analysis, we found that NO appeared to reduce the risk of postoperative AKI in patients undergoing CPB, suggesting that the effect of NO on renal function might be disease specific. Future trials involving NO therapy are required to consolidate our findings and investigate potential mechanisms of renal protection properties.

## Supplementary information

**Additional file 1.** Search strategy.

**Additional file 2.** Risk of bias graph.

**Additional file 3.** Funnel plot for the primary outcome.

**Additional file 4.** Forest plot for the Peto method. OR, odds ratio. CI, confidential interval; AKI, acute kidney injury; NO, nitric oxide.

**Additional file 5.** Forest plot for sensitivity analysis including studies that had lower risk of bias and reported AKI using KDIGO criteria. RR, risk ratio. CI, confidential interval; AKI, acute kidney injury; NO, nitric oxide.

**Additional file 6.** Forest plot for the length of ICU stay. MD, mean difference. SD, standard deviation; CI, confidential interval; NO, nitric oxide.

**Additional file 7.** Forest plot for the length of hospital stay. MD, mean difference. SD, standard deviation; CI, confidential interval; NO, nitric oxide.

**Additional file 8.** Forest plot for the risk of postoperative hemorrhage, i.e., requiring blood transfusion after the operation or reoperation. RR, risk ratio. CI, confidential interval; NO, nitric oxide.

**Additional file 9.** Forest plot for levels of methemoglobin at the end of CPB, %. MD, mean difference. SD, standard deviation; CI, confidential interval; NO, nitric oxide; CPB, cardiopulmonary bypass.

## Data Availability

The datasets used in this study are available from the first author or corresponding author on request.

## Abbreviations

AKI: Acute kidney injury
ARDS: Acute respiratory distress syndrome
CPB: Cardiopulmonary bypass
CI: Confidence interval
Hb: Hemoglobin
ICU: Intensive care unit
MetHb: Methemoglobin
NO_2_: Nitrogen dioxide
MD: Mean difference
RR: Relative risk
RCT: Randomized controlled trial
RRT: Renal replacement therapy
RBCs: Red blood cells
TSA: Trial sequential analysis
RIFLE: Risk, injury, failure, loss and end of stage
AKIN: Acute kidney injury net
KDIGO: Kidney disease: improving global outcomes
