# Supplementary material for: Effect of nitric oxide on postoperative acute kidney injury in patients who underwent cardiopulmonary bypass: a systematic review and meta-analysis with trial sequential analysis
Source: Ann Intensive Care. 2019 Nov 21;9:129. doi: 10.1186/s13613-019-0605-9 (PMC6872705; doi:10.1186/s13613-019-0605-9)
Supplement: Supplementary file 1 — Additional file 1. Search strategy. [file 13613_2019_605_MOESM1_ESM.docx]

Embase: 2327

('cardiopulmonary bypass'/exp OR 'systemic pulmonary shunt'/exp OR 'cardiopulmonary bypass':ab,ti OR 'heart-lung bypass':ab,ti OR 'heart lung bypass':ab,ti OR 'heart-lung bypasses':ab,ti OR 'cardiopulmonary bypasses':ab,ti OR fontan:ab,ti OR 'mitral valve':ab,ti OR 'cardiac surgery':ab,ti OR 'lung transplantation'/exp OR 'lung transplantation':ab,ti OR 'lung grafting':ab,ti OR 'heart surgery'/exp) AND ('nitric oxide'/exp OR 'nitric oxide':ab,ti) AND ('randomized controlled trial'/exp OR 'controlled clinical trial'/exp OR randomized:ti,ab OR placebo:ti,ab OR 'drug therapy':lnk OR randomly:ti,ab OR trial:ti,ab OR groups:ti,ab)

Pubmed: 535

("Cardiopulmonary Bypass"[Mesh] OR Cardiopulmonary Bypass[tw] OR Heart-Lung Bypass[tiab] OR Heart Lung Bypass[tiab] OR Heart-Lung Bypasses[tiab] OR Cardiopulmonary Bypasses[tiab] OR "Lung Transplantation"[Mesh] OR Lung Transplantation*[tiab] OR Lung Grafting*[tiab] OR Fontan [tiab] OR “mitral valve”[tiab] OR “cardiac surgery”[tiab]) AND ("Nitric Oxide"[Mesh] OR "Nitric Oxide"[tiab]) AND ("randomized controlled trial"[pt] OR "controlled clinical trial"[pt] OR randomized[tiab] OR placebo[tiab] OR "drug therapy"[sh] OR randomly[tiab] OR trial[tiab] OR groups[tiab])

Web of science: 2344

('cardiopulmonary bypass' OR 'systemic pulmonary shunt' OR 'cardiopulmonary bypass' OR 'heart-lung bypass' OR 'heart lung bypass' OR 'heart-lung bypasses' OR 'cardiopulmonary bypasses' OR fontan OR 'mitral valve' OR 'cardiac surgery' OR 'lung transplantation' OR 'lung transplantation' OR 'lung grafting') AND ('nitric oxide' OR 'nitric oxide') AND ('randomized controlled trial' OR 'controlled clinical trial' OR randomized OR placebo OR 'drug therapy':lnk OR randomly OR trial OR groups)

CENTRAL: 154


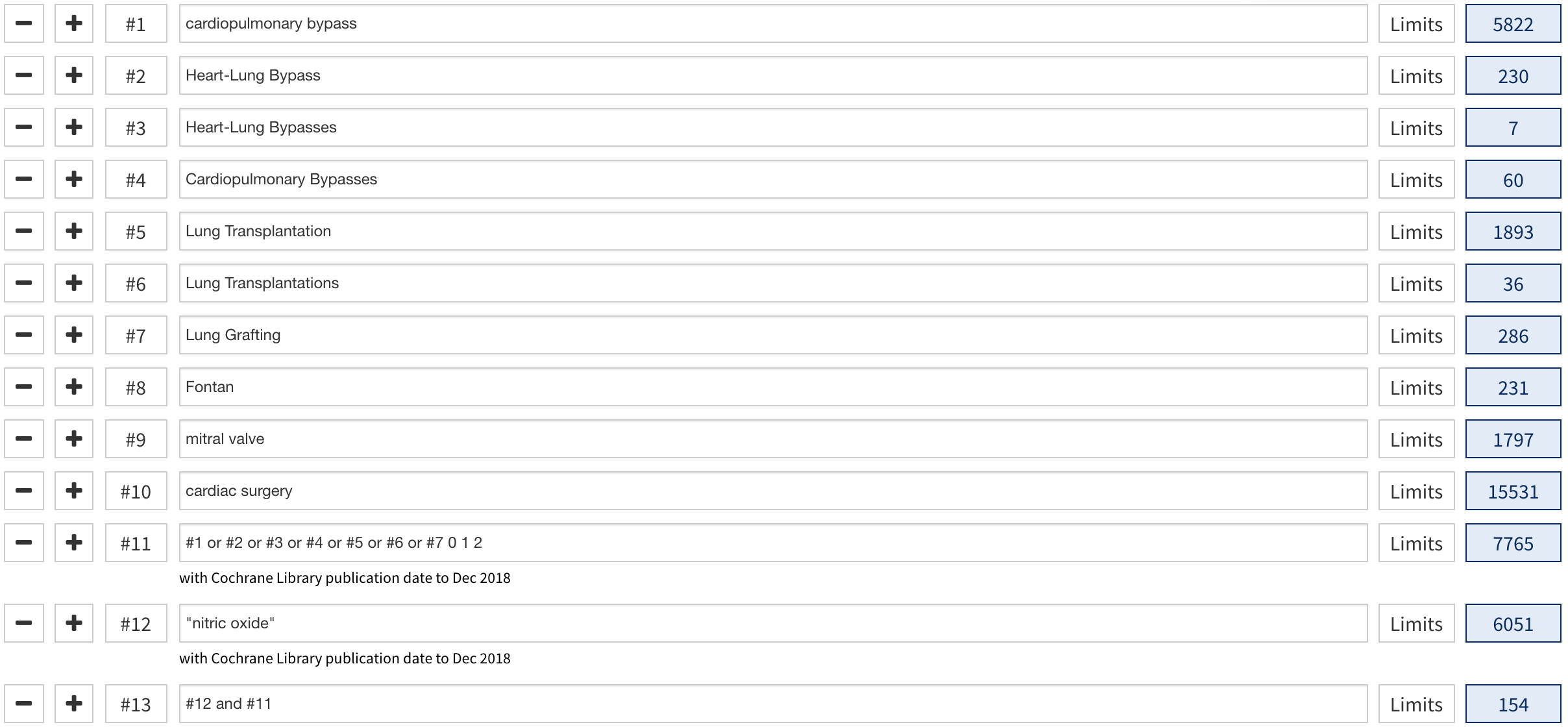


ClinicalTrial.gov: 16

Nitric oxide and cardiopulmonary bypass
