# Supplementary figures and images for: Effect of nitric oxide on postoperative acute kidney injury in patients who underwent cardiopulmonary bypass: a systematic review and meta-analysis with trial sequential analysis
Source: Ann Intensive Care. 2019 Nov 21;9:129. doi: 10.1186/s13613-019-0605-9 (PMC6872705; doi:10.1186/s13613-019-0605-9)

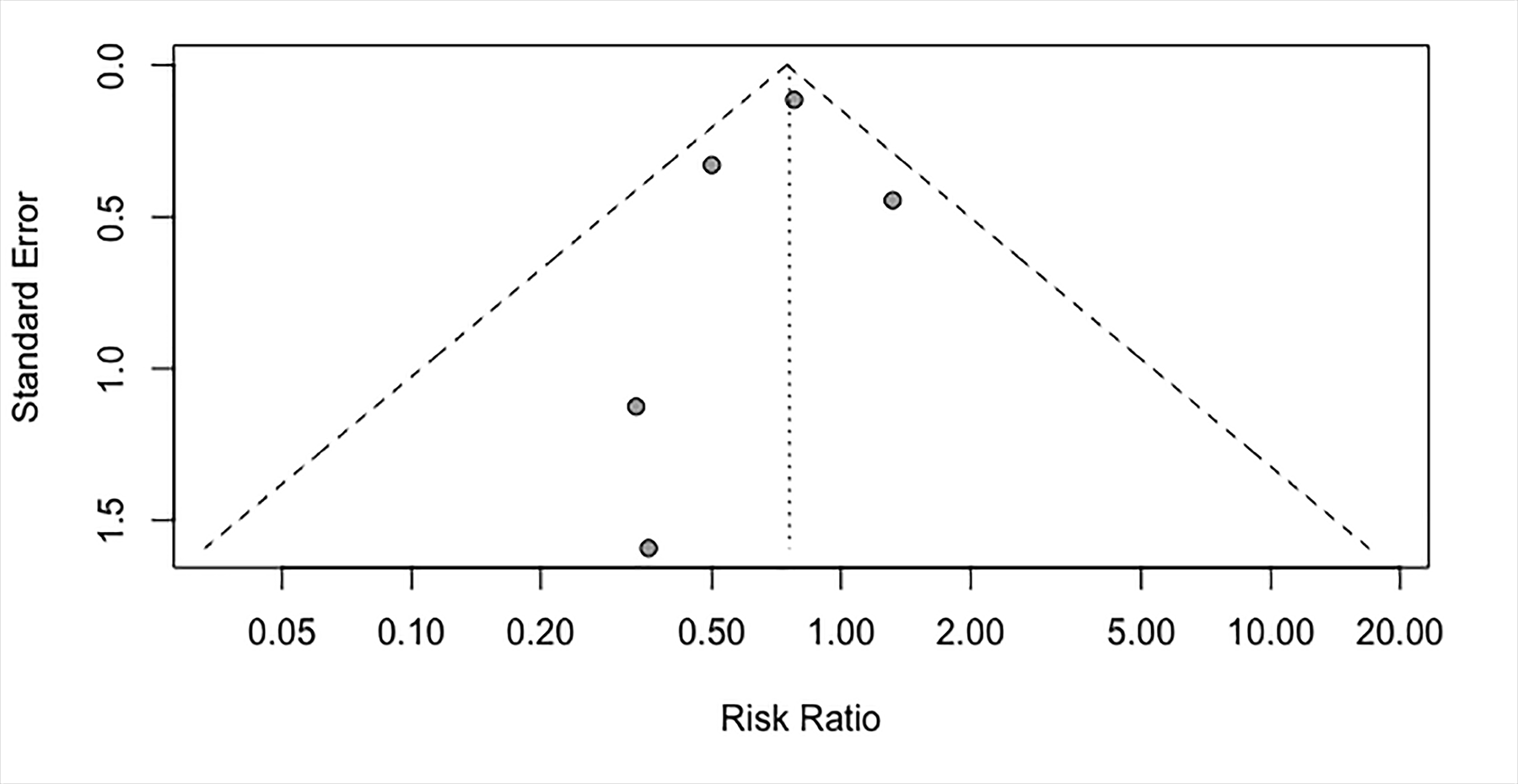

Supplement: Supplementary file 3 — Additional file 3. Funnel plot for the primary outcome. [file 13613_2019_605_MOESM3_ESM.tif]

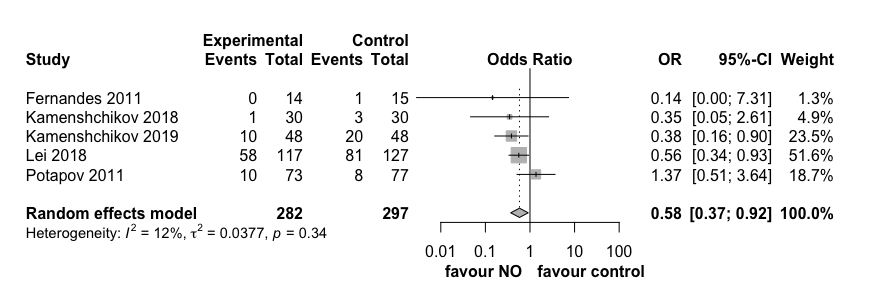

Supplement: Supplementary file 4 — Additional file 4. Forest plot for the Peto method. OR, odds ratio. CI, confidential interval; AKI, acute kidney injury; NO, nitric oxide. [file 13613_2019_605_MOESM4_ESM.tif]

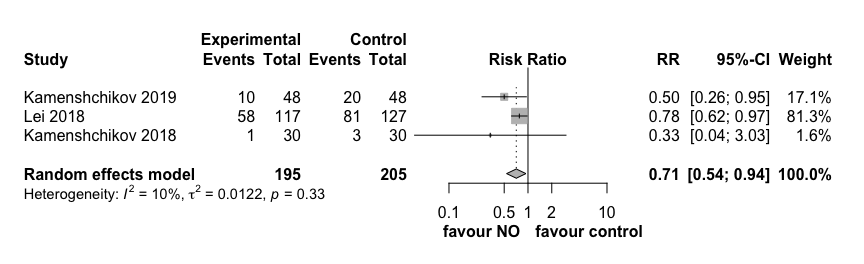

Supplement: Supplementary file 5 — Additional file 5. Forest plot for sensitivity analysis including studies that had lower risk of bias and reported AKI using KDIGO criteria. RR, risk ratio. CI, confidential interval; AKI, acute kidney injury; NO, nitric oxide. [file 13613_2019_605_MOESM5_ESM.tif]

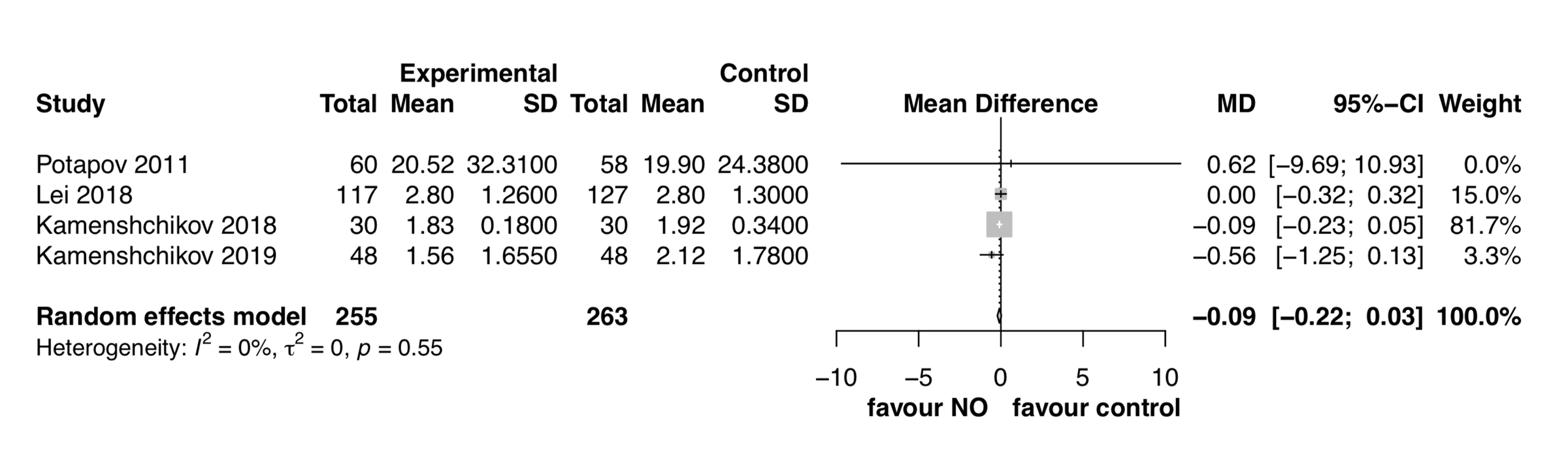

Supplement: Supplementary file 6 — Additional file 6. Forest plot for the length of ICU stay. MD, mean difference. SD, standard deviation; CI, confidential interval; NO, nitric oxide. [file 13613_2019_605_MOESM6_ESM.tif]

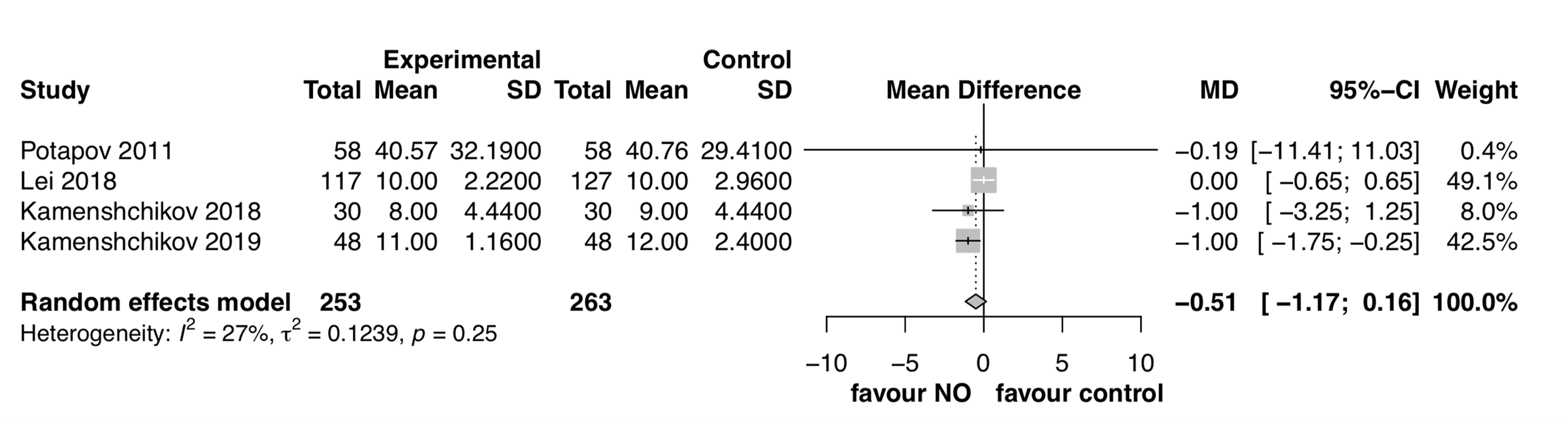

Supplement: Supplementary file 7 — Additional file 7. Forest plot for the length of hospital stay. MD, mean difference. SD, standard deviation; CI, confidential interval; NO, nitric oxide. [file 13613_2019_605_MOESM7_ESM.tif]

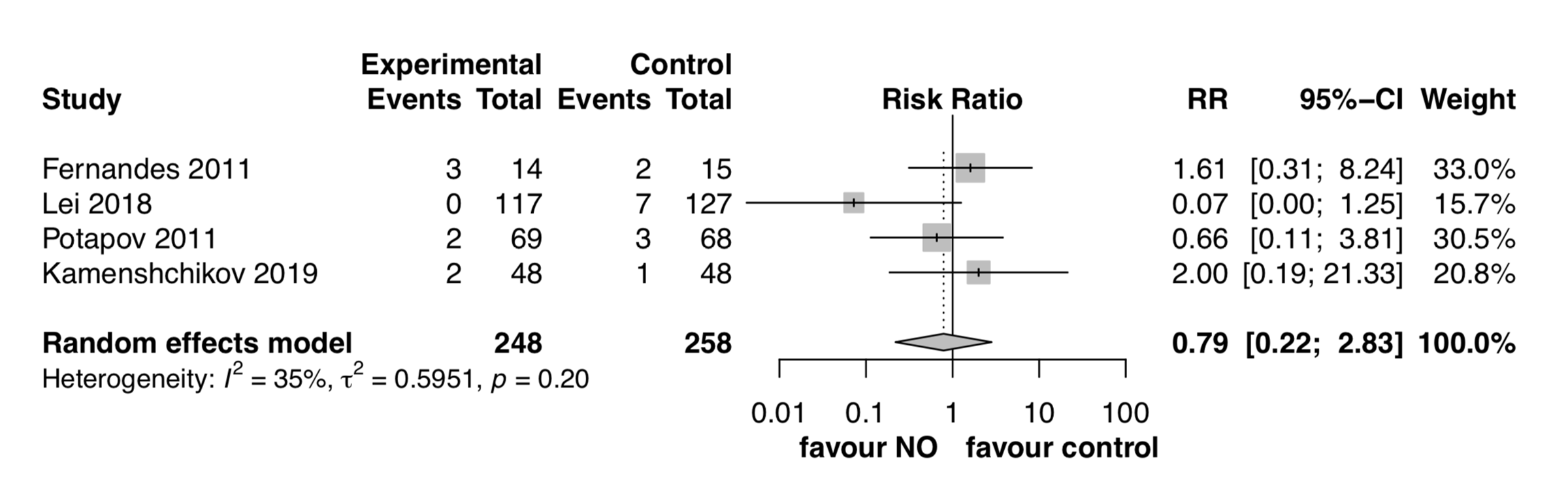

Supplement: Supplementary file 8 — Additional file 8. Forest plot for the risk of postoperative hemorrhage, i.e., requiring blood transfusion after the operation or reoperation. RR, risk ratio. CI, confidential interval; NO, nitric oxide. [file 13613_2019_605_MOESM8_ESM.tif]

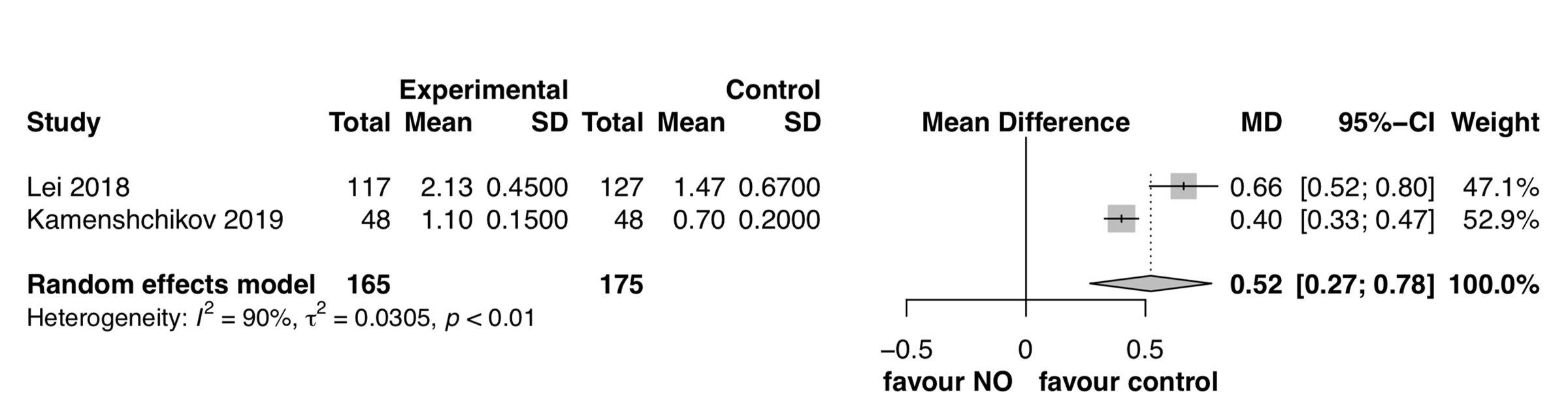

Supplement: Supplementary file 9 — Additional file 9. Forest plot for levels of methemoglobin at the end of CPB, %. MD, mean difference. SD, standard deviation; CI, confidential interval; NO, nitric oxide; CPB, cardiopulmonary bypass. [file 13613_2019_605_MOESM9_ESM.tif]
